# The impact of engaging with community groups: asset-based approaches and the lived experience of socially vulnerable populations in the UK

**DOI:** 10.3389/fpubh.2023.1156422

**Published:** 2023-07-18

**Authors:** Oonagh Corrigan, Suzanne Hughes, Scott Danielsen, Shannon Doherty, Russell Kabir

**Affiliations:** ^1^Faculty of Health, Education, Medicine and Social Care, Anglia Ruskin University, Chelmsford, United Kingdom; ^2^Wellbeing and Prevention Team, Colchester City Council, Colchester, United Kingdom

**Keywords:** community assets, social determinants of health, health inequalities, wellbeing, resilience, COVID-19

## Abstract

**Introduction:**

Asset-based approaches to improve citizen wellbeing and address inequalities are now being adopted by public health practitioners. There is some evidence that participatory approaches and the promotion of community assets have the capacity to mitigate against the social determinants that generate health disparities. However, questions remain about how they work in practice. This paper reports on research carried out to provide insight into how a diverse range of community assets support (or not) the wellbeing of vulnerable citizens and to provide an improved understanding of people's lived experiences including the challenges of citizens who belong to community assets face. Two subsequent studies were undertaken in a region of England comprising of two neighboring municipalities where health inequalities are stark. Both municipalities contain within them areas of social deprivation. The initial study was completed in March 2020, 1 week before England's first COVID-19 lockdown restrictions were introduced and the subsequent study was conducted to explore the impact of social restrictions on the community assets forum members.

**Methods:**

A combined phenomenological and ethnographic methodological approach was adopted for both studies. For the initial study ethnographic methods were used including 42 qualitative face-to-face interviews focusing on the lived experience of participants. Observations, informal conversations, photographs, and field notes were also carried out to allow researchers to become familiar with the setting, to build rapport and trust provide a contextual understanding of the relationship between the activity or place and participants' experiences of wellbeing. For the subsequent study thirty-six interviews (including interviews with community assets leaders) were conducted online or by phone due to COVID-19 social restrictions.

**Results and discussion:**

The studies' findings support the supposition that these groups are community assets which help ameliorate against the social detriments of health and wellbeing that have led to widening health inequalities in the region. The findings from both studies clearly illustrate the importance of sociality for wellbeing, and that participation in these groups are an important determinant of wellbeing. The data demonstrates how social capital is generated within a range of community groups and spaces. It also conveys the needs and deficits existing within groups highlighting the need to provide more assistance to vulnerable citizens. While most themes were common to all community forums, there were some noticeable place-based differences.

## Introduction

Health inequalities are a worldwide problem caused largely by socio-economic disparities:

In all countries—whether low-, middle-, or high-income—there are wide disparities in the health status of different social groups. The lower an individual's socio-economic position, the higher their risk of poor health (1).

The need to provide appropriate health care in the context of growing health inequalities and limited financial resources is faced in many countries across the globe. The two main measures of health inequality are life expectancy and socio-economic circumstances (2). In his recent review of health inequalities in England, Michael Marmot found inequalities have continued to grow, rises in life expectancy have stalled, and that there is a growing regional divide in health, with many regions being “left behind” (3). Epidemiological research has long provided insight into the social determinants of a given geographical area, identifying population deficits and needs. However, there is a mounting critique that this approach while conveying prevailing deficits, fails to recognize the resources that communities and individuals possess to create and maintain health and wellbeing (4).

As a result, new asset-based approaches to wellbeing are now being adopted by public health practitioners with the aim of benefiting the overall health and wellbeing of citizens (5). There is growing recognition that social capital can benefit the wellbeing of citizens and that a demise of this in modern societies has been detrimental (6). Putnam's (7) influential study epitomizes “bowling alone” as an example of this effect representing a shift away from social interactive activities to more privatized individualistic ones. The WHO recognize that community engagement and empowerment are essential components with which to addresses the social determinants of health and help ensure fair decision-making on health equity issues across the globe (8). Marmot's review of health inequalities identifies asset-based approaches as key to reducing health inequalities (3). Public Health England has also endorsed asset-based approached as a strategy to reduce health inequalities (9). There is some evidence that community and individual assets can facilitate people's resilience to manage life's challenges and adversity factors that can otherwise lead to individuals withdrawing from social life and experiencing loneliness and social isolation (4, 10). A recent literature synthesis (11), on how community assets could improve the health of people with long term conditions, highlights their capacity to promote people's capabilities, transform attitudes and values, empower individuals and communities, and raise self-esteem and resilience. However, despite a growing body of work suggesting that participatory approaches and the promotion of community assets have the capacity to mitigate against the social determinants that generate health disparities, questions remain about how they work in practice (12). There are gaps in the evidence such as the identification of mechanisms by which assets contribute to health and wellbeing, or the kinds of social action and practice that best grow and sustain individual and neighborhood assets (13).

The authors were commissioned to conduct this research by a recently formed regional alliance organization that brings together local municipalities in North East Essex (Colchester and Tendring) with other commissioners and providers of health and wellbeing services, including local National Health (NHS) and Social Services, as well as third sector organizations to work collaboratively to tackle ill-health and its determinants. One of their main aims is to reduce inequalities and create healthy, resilient communities.

### Study aims

The commissioners asked the research team to provide insight into how a diverse range of community assets support (or not) the wellbeing of vulnerable citizens; to identify what factors enable community groups and their members to thrive or not, and to provide an improved understanding of people's lived experiences and the challenges they face.

## Methods

### Design

The study brief was to utilize qualitative research methods as this type of research explores and provides deeper insights into real-world problems. The initial study was carried out between October 2019 and June 2020 with data collection being completed in March 2020, 1 week before England's first COVID-19 lockdown restrictions were introduced. We were asked to undertake a further study to gain insight into how these groups and their members had experienced the COVID-19 pandemic.

The subsequent study was conducted between November 2020 and August 2021 and expanded to include a sample of the original participants and inclusion of two groups from minority ethnic populations who were understood to be particularly vulnerable during the COVID-19 pandemic (14). A combined phenomenological and ethnographic methodological approach was adopted for both studies.

### Setting

The research was undertaken in North East Essex, comprising two neighboring municipalities situated in the East of England where health inequalities are stark. Both municipalities contain within them areas of social deprivation although while in Colchester (area 2) there are pockets of deprivation, in Tendring (area 1) deprivation is more widespread.

Municipality 1 (Tendring) is a coastal and rural region where the general health of citizens is worse than the England average. Its population size has increased by 7.3% since 2011 and currently stands at 148,100 in 2021 (15). This is higher than the overall increase for England (6.6%). There are numerous areas of social deprivation in Tendring where vulnerable populations include a high percentage of older people and low-income families. Clacton, for example, is a coastal town which has areas of high deprivation (16). A recent report found Clacton to be one of England's “Left behind” neighborhoods characterized by poor social infrastructure (17). In 2019 the nearby suburb of Jaywick was once again identified as the most deprived area in England (17). Also, Harwich is a port town ranked in the top 10% of the most socially deprived areas of England and is poorly connected to other parts of Tendring. Overall, life expectancy in Tendring is 10.6 years lower for men and 7.8 years lower for women in the most deprived areas than in the least deprived areas (16).

Municipality 2 (Colchester) is an area encompassing a small-to- mid-sized city and surrounding suburbs. The population size has increased by 11.3% since 2011 making it one of the fastest growing places in England with a population size currently at around 193,000 (18). While the social and economic demographic profile is more mixed overall, there are pockets of extreme social deprivation and specific issues relating to higher-than-average violent crime rates and a large transient population (19). In 2019 life expectancy in this region was 8.6 years lower for men and 8 years lower for women in the most deprived areas of Colchester than in the least deprived areas (19).

### Sample

Drawing on local and national government statistical data to identify areas of social deprivation and local demographic and place-based information, a purposive sampling method (20), was adopted to identify community forums consisting largely of socially vulnerable populations. This data included health profiles, age, and other community assets mapping indices which identified many of the community groups in the region. Given that the definition of community assets includes social and support groups and community spaces/places, six community assets (3 in each municipality) were identified composed of 5 community/support groups and one community hall. See Tables 1, 2 for further information on activities and frequency of gatherings. In the initial study, 42 members took part in interviews (see Tables 1, 2). For the second study a sub sample (12 of the original participants) and 12 additional participants from two black and minority ethnic (BAME) community groups were interviewed. The additional groups were added as minority ethnic populations were poorly represented in the initial study and there was recognition of the poor health and wellbeing outcomes during the pandemic (21). Finally, leaders/conveners from all eight assets were interviewed to gain further insights into the challenges involved in reconvening groups after COVID-19. A topic guide was developed and used for all interviews as an aid to direct conversations and to elicit a narrative account of participants' experiences.

**Table 1:** Community groups, settings, and interviewees—Municipal area 1.

**Organizations**	**Description**	**No. interviews in study 1**	**No. interviews in study 2 (leaders in brackets)**
Dementia Café	Run by Community Voluntary Services, established as a place for people with dementia and their families to meet socially and to share information. Provides weekly meetings and free transport to and from the venue.	6	3 (1)
Teentalk	Run by a charity offering a free and confidential information and support service and activities to address the emerging signs of mental health and life challenges faced by young people aged 11–25. Support and activities available throughout the week.	8	3 (2)
Indoor Bowls Club	A self-funded club run by volunteers, providing indoor bowling rinks and matches. Facilities include and a club bar serving meals and drinks. The club aims to be inclusive facilitating games that are mixed men/women and people with disabilities. It is open seven days a week and has more than 45 members aged mostly over 65 years.	6	3 (1)

Municipal area 1 (Tendring)—Community groups for older people and young adults living in the most deprived coastal areas.

**Table 2:** Community groups and settings and interviewees—Municipal area 2.

**Organizations**	**Description**	**No. interviews in study 1**	**No. interviews in study 2 (leaders in brackets)**
Project Nova	Run by a regional charity supporting veterans who have been arrested or are at risk of police arrest. Staff and volunteers help support veterans integrate back into civilian life and keep in touch with them to provide ongoing support, checking in and resolving issues when they occur. Support is available daily with occasional group activities.	8	2 (1)
Uniform Exchange	Run by a Christian communities-based charity supporting people in need by providing the exchange of second-hand children's school uniforms and advice about financial support for people on low incomes. The Project is partnered with a local Foodbank and provides uniform exchanges in local community venues.	7	1 (1)
Community halls in partnership project:	This co-operative project enables several community halls to develop new ways of working collaboratively with the aim of forming a “network” of appropriate and sustainable community buildings.		(1)
1. Local MS Society	This is a local group which is part of a national charity, supported by volunteers providing friendship, support and information about Multiple Sclerosis. Weekly meetings.	2	(1)
2. Friendship Group	Self-funded by members to meet weekly, share lunch and participate in various activities such as quizzes.	3	3 (1)
3. Parent and Toddler Group	Self-funded, provides a weekly meeting space for toddlers to play and interact (from birth to 3 years) and parents to socialize with others. It is led by a trained volunteer.	2	(1)
Fresh Beginnings	A voluntary sector organization with employed key workers and volunteer befrienders providing practical help to support refugees settle in and integrate into the local community.	Not included in first study	4 (1)
Islamic Community Center	A registered charity that was established to meet the needs of local Muslims providing a community center hub for local Muslims and a place for connecting with all communities in the city.	Not included in first study	4 (2)

Municipal area 2 (Colchester). A sample of community assets consisting of community groups and places. These include a cooperative of community halls selected in the more socially deprived areas and a community of veterans. Later, a community of newly settled refugees was chosen to reflect the transient population.

### Data collection

Ethnography, which explores people's actions in a given social context and their own interpretation of such behavior (22) was combined with a phenomenological approach that foregrounds people's “lived experience” (23). This “life-world”-analytical ethnographic mixed methods approach supports validation of findings as they both seek to elicit meaning in the narrative accounts of research participants (24, 25). These methods allow research to capture the significance of community assets for people's everyday lives and to situate this in the context of the communities where they live. Drawing data from various sources reduced the chances of reaching false conclusions (26).

For the initial study ethnographic methods were used including informal conversations, field notes, taking photographs of places, and people engaged in activities, and qualitative interviews focusing on the lived experience of participants. Observations, informal conversations, photographs, and field notes were carried out to allow researchers to become familiar with the setting, to build up rapport and trust with group leaders and members and provide a contextual understanding of the relationship between the activity or place and participants' experiences of wellbeing. In-depth interviews with members (both studies) and with leaders (study two) were carried out. These were conducted in person for the initial study and due to prevailing COVID-19 social restrictions, conducted online or by phone in the second study.

#### Analysis

Interview recordings were transcribed and coded using a descriptive framework. Subsequently, common themes were identified using established thematic analysis techniques (27). The analysis was informed both by issues and concepts that stem from the research topic, such as identification of resources (28) and capabilities (29) enabling some citizens to thrive (or not) in challenging situations, and elements that were discerned in the data, such as broad categories, repeated words, phrases, understandings and experiences present (30). To ensure internal validity, lead researchers carried out coding and thematic analysis of all transcripts. A third member of the research team read and analyzed a sample of transcripts, and the study lead provided a final level of analysis including sub-themes. Findings were discussed further by the whole team. Observation notes and photographs were drawn upon to provide further contextual insight into the asset and to help enhance validity for interpretation of the interview data.

#### Ethics and consent

Prior formal ethical approval for each study was granted by Anglia Ruskin University's School of Allied Health, Nursing, Midwifery, and Medicine's Research Ethics Panel (reference AH-SREP-19-044) and the Faculty of Health, Education, and Medicine and Social Care' Ethics Panels (reference FREP 20/21/011), respectively. Access to participants was facilitated by relevant gatekeepers (those leading/organizing the assets). Observations, photographs, and interviews were conducted when participants had freely given their prior written informed consent. Pseudonyms were assigned to all study participants although some names of assets organizers are genuine as they did not wish for anonymity.

## Results

### Sociality

One of the strongest themes identified was sociality, the value participants placed on the everyday life world as a realm shared with others. The term “sociality” broadly refers to the sociability of people and their tendency to associate with others and to form social groups (31). Here it is deployed to refer to the process of relating to others through actions such as making and being friends with others, forming a sense of belonging, and sharing activities and information.

#### Friendships: connecting and caring

Making new friends was important to Carol who had moved to the coastal town a few years earlier.

I've got a really nice... friends I'd meet up with them and come and play on a Tuesday for a “roll up” [game of bowls] and we go out for coffees …everyone is so friendly and …everyone speaks to you (Carol, Bowls Club).

Other members said the Bowls Club provides them with much more than a game of bowls.

I don't just come down for the games I will socialize, have a cup of tea and whatever (Henry, Bowls Club).

The welcoming atmosphere and companionship at the Club were greatly valued by all the Bowls Club participants.

Everyone in this club has got a bowls family …everyone pushes the chairs out so you're in one huge circle … no one is left out and you don't feel isolated (Sally, Bowls Club).

For Derek who has early-stage dementia, the friendships he made at the club clearly had a positive impact on his mood and sense of wellbeing.

A sense of joy, a sense of … understanding and just a “hello” or “hi how are you?” is very meaningful. We just sang happy birthday and it's just I'm making the best of it (Derek, Dementia Café).

For Colin, meeting other people is what he most valued about the Café.

The meeting of other people. Usually the people you know, …Pam [Dementia Café Leader} a lovely lady, she always introduces new people to me. “Sit next to Colin. You'll get on with Colin” (Colin, Dementia Café).

Like the Dementia Café, the Friendship Group meets once a week and provided much valued social interaction for its older members many of whom lived alone.

I get enjoyment and I think it uplifts you to listen to other people, mainly retired people I do enjoy it (Eileen, Halls—Friendship Group).

Although the principal means of support for the veterans, who are members of Project Nova, is in the form of one-to-one meetings/conversations with the community organization's leader and trained staff, connecting with other veterans was also important to them. Many members participated in the monthly breakfast meetings at the local pub and in other group activities. As Jack said, “it's how we survive.” Project Nova participants enjoyed being part of a group and expressed feelings of camaraderie with other members. I just need to get out there and be something, be part of something (Gary, Project Nova).

Other community group participants also spoke of the friendships they formed with group organizers and volunteers and how this made them feel valued and cared for. Many members who attended the weekly MS forum faced daily struggles living with the condition but spoke of the uplifting effect that other members' expressions of concern had on their sense of wellbeing.

I was a bit later than normal and this morning Hazel (another member) rang up and asked if I was going and that made me feel so wonderful to think that she cares (Lorna, Halls—MS Group).

#### Sense of belonging

Camaraderie and a sense of belonging was especially important for many of our participants. This was particularly salient for those such as Fatima who had little contact with their family. When asked what it meant to be part of the CICC Fatima said,

It means I am part of the community, first of all. I am part of something bigger than myself. I feel that I can reach out for help as well as help others … I don't feel lonely (Fatima, CICC member).

This sense of belonging not only supported Fatima's sense of wellbeing but enabled her to help others. Eighty-two-year-old-David who had type 2 diabetes, lived alone in a trailer home and also had very little contact with his family describes how Bowls Club made him feel socially connected and gave his life purpose.

…I wanted to be with people and be in the world sort of thing …it's my second home really …it gives me something to get up and go in the morning for (David, Bowls Club).

Seventy-five-year-old Peter a widower who also lives alone says he is never lonely as he too spends most days at the Club.

Although the Dementia Café convenes just once a week, participants also found that coming along to the group helped them feel less isolated.

Talking to other people in the same situation—you think you're alone and then you come in and you get chatting and you realize you're not alone. … Dementia Café …was our lifeline [without it] well, I think Mum could have had a breakdown really (Fern, Dementia Café—daughter).

The veterans participants who were supported by Project Nova, told us how they felt valued and respected by Project Nova leaders and this increased feelings of self confidence and self-esteem. A sense of belonging was something veteran participants found challenging since leaving military service and they spoke of how the support they received from Project Nova was helping them to adjust to civilian life.

#### Sharing information

An additional element of connecting with others was that groups provided opportunities to share information and experiential knowledge which was greatly valued by members.

They talk about their prostate problems, and they say well “I've got this,” …and they all talk about it here. Before this I've never known men talk about everything like they do here (Sally, Bowls Club).

You can get a certain amount of advice from other people … you can bounce the problems that you have off other people and … understand whether or not other people are suffering in the same way that you are (Timothy, Dementia Café—husband).

Information about groups and resources available was often shared with people so they could access support which helped them “survive” challenges experienced. Terri heard about the uniform exchange from a homeless charity.

[the children's] biological father had taken his life, things had gone downhill, really bad, we lost our home but thankfully the homeless accommodation found us somewhere to live, they put us in touch with the school uniform and the munch club and bits to get us through (Terri, Uniform Exchange).

Many of the groups not only provided opportunities to share information, but some also supported people in financial need. Jane, a single mother of four school aged children. Spoke about how ordinarily school uniform was an unaffordable expense for her as a single parent on a low income.

I've got 3 skirts (free) and some summer dresses …it's still a struggle when you're on your own (Jane, Uniform Exchange).

As we will reveal in our analysis of how participants experienced COVID-19, sharing information became increasingly important when social restrictions were in place during the COVID-19 lockdowns.

#### Building resilience

Participants described instances on how belonging to a community group provided them with strength and resilience in the face of ongoing life challenges. They spoke of the various hardships they were encountering or had encountered and how belonging to the group helped them build resilience in the face of these challenges.

One of the most important aspects for the Project Nova participants was the strong one-to-one relationships they had with the Group's support team and how this helped them feel calmer and more able to manage their lives. Bill had been referred to Project Nova by a mental health charity following a PTSD diagnosis and concerns about previous suicide attempts. He was being supported by the Project Nova team with regular telephone calls and conversations.

Bill explained the support they gave him encouraged him toward living his life in a better way.

…they point me in the right direction and help me get to that point rather than catch me... they'll give me the destination that I've got to be at and they'll push me at that direction (Bill, Project Nova).

Timothy who cared for his wife who had dementia felt he would be much more stressed if he did not get support from the group.

I think it would increase the stress level of caring. I wouldn't have the information fed back to me that “maybe this is a route that you should be looking at.” … “Why don't you try this? Why don't you try that?” (Timothy, Dementia Café—husband).

Many of the young people we spoke to from Teen Talk described their town as neglected and run down and the group leaders said most young people who came to the group lacked confidence and self-esteem. However, this was something that improved soon after they joined. It was evident that TeenTalk members appreciated the confidence and life skills that the leaders helped them obtain.

If it wasn't for TeenTalk, I wouldn't be as calm as I am approaching people now really.... I was like, “I don't want to do painting at all.” Then now, I don't stop painting because I am somehow convinced …that it's great (Jade, TeenTalk).

Rosie, a member who also helps as a volunteer assisting with Teen Talk's younger members, said her communication skills had improved greatly.

Being able to work with other people and being able to communicate with other people. I think people skills are the most important thing (Rosie, TeenTalk).

Some groups leaders informed us of how several of their members were facing challenging financial circumstances. The leaders of CICC reported seeing an increasing number of their community struggling financially during the pandemic.

…a lot of people have become very hard up financially. …we have been trying to …identify those people and set up a food voucher scheme (CICC).

While most groups did not provide direct financial resources to members, groups often provided advice, with some actively intervening. The leader of the Uniform Exchange Group observed that during the COVID-19 pandemic those on furlough often had similar incomes or less to those on benefits and were not entitled to other benefits such as free school meals.

As such, they're struggling more, …and we've had to …speak to the school and explain the situation and they might be able to help. If not, then we've referred to the food bank …because they are struggling (Uniform Exchange, Leader).

Some participants living in Tendring felt isolated because of a lack of convenient and cheap transport. For example, Bobby who has had to stop driving because of eyesight problems and is also on a low income and finds it a strain both getting to shops where food is cheaper.

…the nearest bus is down the village …is about a half-an-hour walk. …now with the bus company that's taken over the route… you wait for your time, and you wait, and you wait, and you wait (Bobby, Dementia Café).

The free bus service which provides transport to the Dementia Café was an additional help as it enabled Bobby to nip to the supermarket next to the Dementia Café Hall while his wife who has dementia attended the meeting.

Linda felt that she had overcome feelings of stigma and shame associated with being on a low income to go along to the Uniform Exchange venues.

…although my partner works, we do still struggle because we don't get benefits so I would advise people if they want to go to the Uniform Exchange but feel a bit embarrassed to do so …it doesn't make you different …these people are here to help you (Linda, Uniform Exchange).

#### Activities

All groups provided at least some physical and/or mental activities during their meetings which helped participants maintain or gain resilience. The range of activities however varied greatly. For example, the Bowls Club provided the greatest opportunity for physical activity and hosted quiz evenings and days out. Teen Talk provided a range of physical and mental activity opportunities for young people and linked with local charities and organizations to facilitate the learning of new skills, including cookery, painting, gardening, and sailing. Participants at the Bowls Club reported bowling helped them stay physically fit.

I thought I needed a bit of exercise and she [the doctor] said “it would be good for your weight” (Henry, Bowls Club).

Eileen, who attends the Friendship Group, feels that attending weekly group gatherings helps her stay mentally active:

Mixing with different people and talking about different subjects helps you mentally and bodily as well …we've just had a quiz and it taxes your brain and makes you think whereas you wouldn't do if you were just sitting at home (Eileen, Halls—Friendship Group).

Sally who has a long-term health condition told us how she found coming back to the Bowls Club an invaluable part of her physical and emotional recovery from an acute episode.

…I was very poorly. I couldn't even shower myself. I get a bit emotional, I couldn't wash my own hair, my husband had to do everything …I had to have over a year out (from the Club) for illness which is why I'm coming back …this Club has helped me, not only me, my husband and so many other people (Sally, Bowls Club).

#### Life-line

One of the questions we asked members in the first study was “what would life be like if the group did not exist?” Most participants spoke of how vital the group was to their wellbeing, often describing the community group as a “lifeline,” with some stating they felt it was the only thing that had kept them alive. Colin who had been diagnosed with dementia, likened his attendance at the club to that of giving and receiving a blood transfusion. When asked what the club meant to him, he put his arm out as he said:

…the club is my lifeblood. That's the way I look at it. I'd put my arm and have blood taken or given. I look forward to it so much every week. I really do (Colin, Dementia Café).

Lorna, who has MS and lived alone had been attending the MS Group for 8 years, said “…this has been a life-line for me.”

All the veterans we interviewed felt that without Project Nova they would unlikely have survived daily life.

I would have committed suicide, I'd have checked out long ago (Harry, Project Nova).

I would have either been dead or in jail. I probably wouldn't have gone to jail. …I couldn't have coped with it and probably done myself in (Stan, Project Nova).

The hypothetical situation of groups closing became a grim reality for many when COVID-19 restrictions were introduced in March 2020.

### Impact of COVID-19

There was great variation in the level of support provided for members during the pandemic. While for some groups the support given to members by leaders and volunteers was extensive and involved ongoing interactions with members such as regular phone calls, online contact and involving them in projects and activities, for others, the support was less frequent and more *ad-hoc*.

#### Social isolation and mental wellbeing

Unsurprisingly, some of our participants reported feeling very socially isolated, particularly older members, those who were having to shield for a longer period during the pandemic due to health, age, or disability status, and those living alone. The Dementia Café members were the most severely adversely affected. The closure of the Café due to the pandemic meant that for those living with dementia and their carers were left feeling isolated and vulnerable.

We're a bit lost because Wednesday café was the only time, we went out each week. …The café was a lifeline before because we had a definite thing to do every Wednesday (Bobby, Dementia Café).

Ellen found that her husband's behavior became very challenging to deal with when the café closed.

He became very withdrawn …very, very moody; very snappy; and he took all his anxiety and anger out on me …he really loved the café, and it did cause a huge blow to him (Ellen, Dementia Café).

He became increasingly frustrated as time went on and in the end his behavior led Ellen to experience a mental “breakdown” episode.

I had a bit of a breakdown, and social services and the Admiral Nurses got involved and I just lost it 1 day with one of the nurses. I was just like a babbling mess really. I just couldn't cope. He had to go into respite, because it took longer than 4 weeks for me to recover (Ellen, Dementia Café).

Two of the three Dementia Café participants we followed up for the second study reported they had experienced a “breakdown.”

The Dementia Café leader told us that carers really suffered because of the café closure during the pandemic.

They've gone from some of them literally only having that one thing [weekly Café meetings] to having nothing other than a phone call, which doesn't seem like very much support to be honest in the long run for everybody (Dementia Café, Leader).

As the Indoor Bowls Club members were also older in age many of the members who lived alone said that they felt isolated and lonely without the club during COVID-19 restrictions.

Eighty-three-year-old David who lived alone in a mobile home was particularly unhappy as he had relied on the club for company and his meals before the lockdown.

It hasn't been very nice living on my own. …Not being able to go out and mix with people and stuck in one room (David, Bowls Club).

Ninety-three- year-old Bertha from the Friendship Group club said she felt “alone and isolated.” Although she received weekly visits from her daughter, she missed the companionship of other Club members and said that she had left the house on just one occasion since lockdown began. She experienced a fall at home which left her feeling vulnerable.

Eighty-three-year-old Betty, another Friendship Group member living alone, reported how she felt increasingly isolated as the year progressed.

The first lockdown. I used to sit outside and talk to my neighbors. Then I'd go around the block for a walk. As it got colder, I just stopped in. …I never go anywhere. …It just got to me. It really got to me (Betty, Friendship Group).

While experiences of social isolation were predominantly reported by older participants, concern was also expressed about the effects of social restrictions on younger people in relation to loss in confidence, and increased anxiety.

The toddler Group did not meet for more than 1 year and the Group leader said that some parents were concerned that a lack of social interaction with others was adversely affecting toddlers' development.

I have had messages from some of the mums saying … “we've now got a younger child who literally has not learned to socialize with other children (Parent and Toddler, Leader).”

It was clear that some participants were struggling with their mental health. David, a Bowls Club member, had suffered a mental breakdown when he was younger and was concerned that having to stay in could very easily trigger “another breakdown.”

Some of the refugee participants supported by Fresh Beginnings reported feeling isolated and lonely.

I was feeling lonely, and the lockdown prevented me from visiting my family. In the time of coronavirus, the world has changed into it is like a ghost town. I became depressed and scared… my condition was very bad due to isolation restrictions (Amara, Fresh Beginnings).

The MS Group leader said the group's members were all shielding and had greatly missed face-to-face contact and that shielding had been detrimental to members' mental wellbeing.

They definitely miss it. We've been almost locked away. It's doing people's heads in. I'm the same I haven't been out for over a year (MS Group, Leader).

Veterans also experienced increased anxiety as lockdown heightened their PTSD symptoms.

I think isolation has been an issue. …with some people. It's been really difficult for them (Project Nova, Leader).

Betty found the social isolation she experienced was having an impact on her mental wellbeing.

…I'm getting to the point now where I can't be bothered, I sit there and still don't put my telly on … I used to think I'm doing really well, and I used to stay active and I've just let myself go (Betty, Friendship Group).

In addition to experiences of loneliness due to the isolation, the pandemic also caused increased feelings of fear and anxiety, especially among some ethnic minority communities who were aware of a heightened risk of contracting coronavirus and being hospitalized or dying of the virus.

[Many experienced] …emotional difficulties, isolation, loneliness. …Many people were very fearful. They were fearful of the disease, and they were fearful of the vaccine. We did our best to overcome that as well and we're in the fortunate position of having many doctors who are members and a good resource for us (CICC, Leader).

#### Loss of mobility during COVID-19

Once again, it was the older participants or those who were shielding, whose physical fitness and mobility were most badly affected.

I seem to have got worse with it. I suppose it is the sitting that's affecting my back. I do try and walk about a bit …you're not moving about so much indoors, whereas you used to get up and get ready and go out (Ellen, Dementia Café).

Dementia Café members caring for their spouses found it very challenging to get out of the house for exercise. Bobby told us how he never managed to leave the house for long or go very far.

The Dementia Café leader reported that many members were experiencing a deterioration in their mobility.

They are having problems with their balance where they haven't been out. They haven't been able to walk as much as they have done. …they are struggling walking …A lot of them have gone quite downhill (Dementia Café, Leader).

Ninety-three-year-old Bertha from the Friendship Group said that after a 4-month period of not getting about at all, her legs “were like jelly.” Betty, another member of the Friendship Group, said she has been experiencing some stiffness, aches, and pains.

The MS Group leader said that his own health had deteriorated during the pandemic but that they had continued to provide online Yoga classes for members.

#### Keeping engaged and active

Some groups engaged their members in activities during lockdown. Colchester Islamic Community Center (CICC) members spoke about activities they were engaged in through the WhatsApp groups and how helpful Zoom lectures were in providing health and wellbeing information during the pandemic.

There was a lot of talk about how important the vaccination is, initially we were a bit hesitant …then listening to the lectures made us want to take it more. [There were] definitely some good talks from the doctors and pharmacists about a variety of things (Laila, CICC).

Laila spoke about how she enjoyed the weekly Tai Chi classes that were organized via Zoom and found the check-in phone calls really helped during lockdown.

TeenTalk members also engaged with a range of digital platforms.

They've still been able to run the groups on Zoom, …They've got groups they can go along to and chat to other people their own age. …I don't obviously see many people at the moment, so it's nice just to see someone (Rosie, TeenTalk).

Online communication was not the preferred way of communicating for Bowls Club members. However, some members kept in touch by telephone and emails and when the club leader discovered that the two members of paid Staff who had previously cooked food at the club's café were cooking and delivering meals from their homes during COVID-19 to support members living alone, he invited them to prepare the meals at the club. Later, when social distancing restrictions permitted, dining facilities were resumed at the club before playing Bowls was permitted.

Teen Talk Leaders tried to ensure that young people kept active during the pandemic, organizing one-to-one walking groups and gardening activities. They and some of the other groups initiated creative projects and activities to help promote positivity, a sense of belonging for members, and strategies reach out to those in need during the pandemic. Teen Talk members put together art activity packs including paints, pens and craft materials and delivered these to over 300 young people across the locality. With these packs, young people were encouraged to make objects containing positive quotes and to leave them for others to pick up and read in their communities.

So far, we've had over 800 young people and adults involved, which has been amazing …we've really branched out across Tendring (TeenTalk, Leader).

The Group also delivered positive post cards to local Care Homes. These ideas for activities were generated by the young people themselves and often volunteer member led.

CICC instigated a variety of different projects throughout to help keep members spirits up and to help those in the wider community. One of the early initiatives was to distribute iftar food parcels for CICC members.

We included in that a bunch of flowers for the ladies and a box of chocolate for the men and it was amazing. … it had made a difference (CICC, Leader).

Samir helped to deliver food parcels to people who were fasting during Ramadan.

We wanted to help the families in Colchester who need help in terms of food for iftar, for when they break the fast, we wanted to give them good food. Last year and this year we helped doctors to give them iftar (Samir, CICC).

The group's members also formed a sewing group and made PPE items such as face masks, which were donated to members and others in the wider community which enhanced members' sense of wellbeing.

We're busy and happier and doing something creative as well, it makes you happy and it gives the confidence as well' (Laila, CICC).

## Discussion

A unique feature of this research is its revelation of the lived experience of members across a range of very diverse community groups in terms of size, frequency of meetings, funding sources, and composition. Despite the heterogeneity of this sample, our findings demonstrate these were forums across both municipalities where members felt heard, safe, and experienced a sense of belonging. So, for example for participants who attended groups that met once a week as well as those who gather less often and those who gathered on almost daily basis, these community forums were a life-line. Sociality, namely connecting with others, forming bonds of friendship sharing activities and information all provided the participants with social capital: an important resource for members and a community asset that can help ameliorate against many of the prevailing detrimental factors in relation to wellbeing. The findings from both studies clearly illustrate the importance of sociality for wellbeing, and that participation in these groups are an important determinant of wellbeing (8). This research adds to the existing body of work on community assets, by demonstrating how social capital is generated within a range of community groups and spaces.

The narrative accounts gathered here also convey the needs and deficits existing within the groups, such as the need to provide more assistance to vulnerable citizens. While most themes were common to all community forums, there were some noticeable place-based differences. Tendring's population is spread over a larger area with some coastal and more rural areas having poor and relatively expensive transport links. The need to improve transport in Tendring (Municipality area 1) was identified as an important issue for older participants as well young people as the population is more dispersed with poor public transport infrastructure. Unsurprisingly, these deficits were amplified during COVID-19. While community assets can help mitigate against the social determinants that generate health disparities, members who had underlying health conditions, older people, carers, those socially disadvantaged such as immigrants, minority ethnic populations, and those living with a disability, were particularly vulnerable to the negative effects that social distancing measures created during the pandemic.

Participants described how these friendships, and feelings of connectedness and belonging enabled them to cope in the face of challenges. Social connectedness is widely recognized to be a predictor of resilience (32, 33). These community assets appear to help individuals build “resilience,” that is, “the ability to successfully adapt to stressful circumstances, and therefore effectively manage stress” [(34), p. 328]. Participants appreciated how much providers, volunteers and members genuinely cared about them. They felt heard, understood, listened to, and valued. This was often in stark contrast to their experiences with more formal statutory health and social services. It was clear that for those who had poor prior experiences with services (this applies particularly though not exclusively to the veterans) this had undermined their trust in statutory services leading to feelings of betrayal, exclusion and anger.

From our observational and interview data we identified an ethos of leaders, volunteers and members being “all in it together” and participants were regarded in a holistic way. The leaders we spoke to identified with other participants, came alongside them and were keen to meet their needs in the context of their everyday lives in non-judgmental ways. The relationship between assets leaders was less based on a service delivery model with “clients” and more one on mutual respect and a common goal. Many participants said that they felt valued and respected and belonging to the community group increased feelings of self-confidence and facilitated their ability to cope with the challenges they faced. This confirms Morgan and Ziglio's (35) claim that community assets operate as protective factors to buffer against life's stresses by promoting salutogenic resources to enable self-esteem and coping abilities of individuals and communities. It was evident that for many participants these community assets were a lifeline.

The community forums not only generated a sense of wellbeing among their members, but in some instances, they supported the wellbeing of others in the wider community. During the pandemic Teen Talk and the Colchester Islamic Community Center members in particular reached out to the wider community by carrying out creative sharing projects. Most groups provided information and sign posting to other organizations to help support their forum members. These are ways in which community assets act as mechanisms for building the “bonding” and “bridging” of social capital (36). Bonding social capital refers to trusting and co-operative relations between people who share social identities (36) and/or geographically defined communities (37, 38), whereas bridging social capital refers to the relational connections with other groups and communities outside these groups.

## Conclusion

One of the benefits of an asset-based approach is that it identifies existing strengths within a community. However, it is important not to lose sight of the ongoing challenges many vulnerable citizens face in the wider municipal areas. Further, it will be vital for local government bodies and health systems to understand the importance of these community assets while recognizing that these assets do not need to be formally institutionalized. To do so may cause community assets to lose their ability to function as community-based entities outside statutory systems where they currently thrive. Our findings reveal strong bonds of social capital within all groups and there is a suggestion that some groups were also engaged in bridging social capital, although there was less evidence of this. However, the further development of bridging social capital would be an important aim in improving the wellbeing of citizens in socially deprived areas.

We suggest there is a need for further research to establish the effects of bridging social capital on wider communities as a means to address social inequalities.

Furthermore, there is a need for local governments and wider health systems to recognize the role that community assets play in creating and maintaining wellbeing and supporting them in this function. Indeed, the findings from this study were warmly received and recommendations made have been used to inform local support for communities and inequality strategies.

Finally, central governments must provide the basis for a fairer more equitable distribution of wealth if inequalities in wellbeing and health are to be truly tackled, as poverty remains the main determinant for health inequality (39). Yet in the absence of a fairer and more equitable distribution of wealth the challenge is left to local governments and health systems to support the development of thriving community assets to buffer against such inequity.

### Study limitations

One of main strengths of this study is the rich descriptive data that provides in-depth insight into the lived experience of participants. However, it would be useful to have collected additional quantitative data on the socio-economic status, ages, and other health related material. Nevertheless, there are inevitable ethical challenges in building good participant rapport and collecting personal health and social class status data from participants.

While qualitative data of this nature is not considered “generalizable,” relatively small scale yet in-depth qualitative studies can mean the findings are “transferable” to other contexts, situations, times, and populations (40).

## Data availability statement

The original contributions presented in the study are included in the article/supplementary material, further inquiries can be directed to the corresponding author.

## Ethics statement

Prior formal ethical approval for each study was granted by Anglia Ruskin University's School of Allied Health, Nursing, Midwifery, and Medicine's Research Ethics Panel (reference AH-SREP-19-044) and the Faculty of Health, Education, and Medicine and Social Care' Ethics Panels (reference FREP 20/21/011), respectively. The patients/participants provided their written informed consent to participate in this study.

## Author contributions

OC led the design of the study, oversaw and carried out data analysis, and wrote the first draft of the paper. SDa contributed to conception of study. SH contributed to study design and carried out initial coding of qualitative data analysis. RK and SDo contributed to statistical data analysis. All authors contributed to manuscript revision, read, and approved the submitted version.

## References

[1] WHO. Health Inequities and Their Causes. (2018). Available online at: https://www.who.int/news-room/facts-in-pictures/detail/health-inequities-and-their-causes (accessed November 14, 2022).

[2] The Kings Fund. Integrated Care Systems Explained: Making Sense of Systems, Places and Neighbourhoods. (2022). Available online at: https://www.kingsfund.org.uk/publications/integrated-care-systems-explained (accessed November 14, 2022).

[3] Marmot M. Health Equity in England: The Marmot Review 10 Years On. (2020). Available online at: https://www.health.org.uk/sites/default/files/upload/publications/2020/Health%20Equity%20in%20England_The%20Marmot%20Review%2010%20Years%20On_full%20report.pdf (accessed November 14, 2022). 10.1136/bmj.m693 32094110

[4] Van Bortel T Wickramasinghe ND Morgan A Martin S. Health assets in a global context: a systematic review of the literature. Br Med J Open. (2019) 9, 23810. 10.1136/bmjopen-2018-023810 30782888 PMC6367963

[5] Russell C. Rekindling Democracy: A Professional's Guide to Working in Citizen Space. Eugene, OR: Wipf and Stock Publishers (2020).

[6] Portela M Neira I el Mar Salinas-Jiménez M. Social capital and subjective wellbeing in Europe: a new approach on social capital. Soc Indicat Res. (2013) 114:493–511. 10.1007/s11205-012-0158-x25724610

[7] Putnam RD. Bowling Alone: The Collapse and Revival of American Community. New York, NY: Simon and Schuster (2000). 10.1145/358916.361990

[8] WHO. Community Engagement: A Health Promotion Guide for Universal Health Coverage in the Hands of the People. (2020). Available online at: https://www.who.int/publications/i/item/9789240010529 (accessed November 17, 2022).

[9] Public Health England. A Guide to Community-Centred Approaches for Health and Wellbeing. (2015). Available online at: https://www.gov.uk/government/publications/health-and-wellbeing-a-guide-to-community-centred-approaches (accessed January 20, 2023).

[10] Centre for Policy on Ageing. Resilience in Older Age. (2017). Available online at: http://www.cpa.org.uk/information/reviews/CPA-Rapid-Review-Resilience-and-recovery.pdf (accessed November 15, 2022).

[11] Blickem C Dawson S Kirk S Vassilev I Mathieson A Harrison R. What is asset-based community development and how might it improve the health of people with long-term conditions? A realist synthesis. Sage Open. (2018) 8:2158244018787223. 10.1177/2158244018787223

[12] Cassetti V Powell K Barnes A Sanders T. A systematic scoping review of asset-based approaches to promote health in communities: development of a framework. Glob Health Promot. (2020) 27:15–23. 10.1177/1757975919848925 31319777

[13] Hopkins T Rippon S. Head, Hands and Heart: Asset-Based Approaches in Health Care. London: Health Foundation (2015).

[14] Public Health England. Disparities in the Risk and Outcomes of COVID-19. (2020). Available online at: https://assets.publishing.service.gov.uk/government/uploads/system/uploads/attachment_data/file/908434/Disparities_in_the_risk_and_outcomes_of_COVID_August_2020_update.pdf (accessed January 11, 2023).

[15] Great Britain Office Office of National Statistics. How the Population Changed in Colchester: 39. (2022). Available online at: https://www.ons.gov.uk/visualisations/censuspopulationchange/E07000071/ (accessed May 19, 2023).

[16] Public Health England. Tendring Local Authority Health Profile. (2019). Available online at: https://fingertips.phe.org.uk/profile/health-profiles/data#page/1/gid/1938132701/pat/6/par/E12000006/ati/101/are/E07000076 (accessed November 14, 2022).

[17] OCSI. Local Trust. Left Behind? Understanding Communities on the Edge. London: England and Wales (2019).

[18] Great Britain Office Office of National Statistics. How the Population Changed in Tendring: 39. (2022). Available online at: https://www.ons.gov.uk/visualisations/censuspopulationchange/E07000071/ (accessed May 19, 2023).

[19] Public Health England. Colchester Local Authority Health Profile. (2019). Available online at: https://fingertips.phe.org.uk/static-reports/health-profiles/2019/e07000071.html?area-name=colchester (accessed November 14, 2022).

[20] Creswell JW Clark VLP. Designing and Conducting Mixed Methods Research. Thousand Oaks, CA: Sage Publications (2017).

[21] Razai MS Kankam HK Majeed A Esmail A Williams DR. Mitigating ethnic disparities in COVID-19 and beyond. Br Med J. (2021) 372:m4921. 10.1136/bmj.m4921 33446485

[22] Hammersley M Atkinson P. Ethnography: Principles in Practice. London: Routledge (1983).

[23] Husserl E. The Crisis of European Sciences and Transcendental Phenomenology: An Introduction to Phenomenological Philosophy. Evanston, IL: Northwestern University Press (1970).

[24] Gray CM. Living in Two Worlds: A Critical Ethnography of Academic and Proto-Professional Interactions in a Human-Computer Interaction Design Studio. (Doctoral Dissertation), Indiana University, Bloomington, IN, United States (2014).

[25] Honer A Hitzler R. Life-world-analytical ethnography: a phenomenology-based research approach. J Contemp Ethnogr. (2015) 44:544–62. 10.1177/0891241615588589

[26] Hammersley M. Questioning Qualitative Inquiry: Critical Essays. Newcastle upon Tyne: Sage (2008).

[27] Braun V Clarke V. Using thematic analysis in psychology. Qual Res Psychol. (2006) 3:77–101. 10.1191/1478088706qp063oa

[28] Antonovsky A. Unraveling the Mystery of Health: How People Manage Stress and Stay Well. San Francisco, CA: Jossey-Bass (1987).

[29] Nussbaum M Sen A. The Quality of Life. London: Clarendon Press (1993).

[30] Edwards R Weller S. Shifting analytic ontology: using I-poems in qualitative longitudinal research. Qualit Res. (2012) 12:202–17. 10.1177/1468794111422040

[31] Collins. Collins English Dictionary—Complete and Unabridged. Digital Edition. (2012). Available online at: https://www.collinsdictionary.com/dictionary/english/sociality (accessed January 20, 2023).

[32] Fuller A McGraw K Goodyear M. Bungy jumping through life: what young people say promotes well-being and resilience. J Psycholog Counsel Schools. (1999) 9:159–68. 10.1017/S1037291100003071

[33] Norris FH Stevens SP Pfefferbaum B Wyche KF Pfefferbaum RL. Community resilience as a metaphor, theory, set of capacities, and strategy for disaster readiness. Am J Community Psychol. (2008) 41:127–50. 10.1007/s10464-007-9156-6 18157631

[34] Maybery D Pope R Hodgins G Hitchenor Y Shepherd A. Resilience and well-being of small inland communities: community assets as key determinants. Rural Soc. (2009) 19:326–39. 10.5172/rsj.351.19.4.326

[35] Morgan A Ziglio E. Revitalising the evidence base for public health: an assets model. Promot Educ. (2017) 14(2_Suppl.):17–22. 10.1177/10253823070140020701x 17685075

[36] Szreter S Woolcock M. Health by association? Social capital, social theory, and the political economy of public health. Int J Epidemiol. (2004) 33:650–67. 10.1093/ije/dyh013 15282219

[37] Hogg MA. Social Identity Theory. In Understanding Peace and Conflict Through Social Identity Theory. Cham: Springer. (2016). p. 3–17. 10.1007/978-3-319-29869-6_1

[38] Proshansky HM Fabian AK Kaminoff R. Place-identity: physical world socialization of the self. J Environ Psychol. (1983) 3:57–83. 10.1016/S0272-4944(83)80021-830203846

[39] Marmot M. Fair Society, Healthy Lives: The Marmot Review: Strategic Review of Health Inequalities in England Post-2010. London: Marmot Review (2010).

[40] Guba EG Lincoln Y. Fourth Generation Evaluation. Thousand Oaks, CA: Sage Publications (1989).

